# The Impact of Mutations in Wolframin on Psychiatric Disorders

**DOI:** 10.3389/fped.2021.718132

**Published:** 2021-10-21

**Authors:** Saira Munshani, Eiman Y. Ibrahim, Ilaria Domenicano, Barbara E. Ehrlich

**Affiliations:** ^1^Department of Pharmacology, School of Medicine, Yale University, New Haven, CT, United States; ^2^Department of Medicine, Frank H. Netter MD School of Medicine, Quinnipiac University, North Haven, CT, United States; ^3^Department of Biostatistics, Yale School of Public Health, New Haven, CT, United States

**Keywords:** wolfram (DIDMOAD) syndrome, schizophrenia, bipolar disorder, WFS1 mutation, mood disorder

## Abstract

Wolfram Syndrome is a rare autosomal recessive disease characterized by early-onset diabetes mellitus, neurodegeneration, and psychological disorders. Mutations in the gene *WFS1*, coding for the protein wolframin, cause Wolfram Syndrome and are associated with bipolar disorder and schizophrenia. This report aims to connect *WFS1* mutations to their impact on protein expression and structure, which ultimately translates to altered cell function and behavioral alterations of an individual.

**Methods:** Published data were used to compile *WFS1* mutations associated with psychiatric symptoms, both in homozygous patients and heterozygous carriers of *WFS1* mutations. These mutations were evaluated *in silico* using SNAP2, PolyPhen-2, and PROVEAN to predict the effects of sequence variants. Statistical analysis was performed to assess the correlation between the locations of the mutations and the damage prediction scores.

**Results:** Several mutations, clustering in the center and C-terminus of the *WFS1* polypeptide, such as A559T and R558C, are found in individuals with psychiatric diseases and appear particularly impactful on protein structure. Our analysis showed that mutations in all regions of wolframin were present in patients with schizophrenia whereas only cytoplasmic and ER luminal mutations were reported in patients with manic episodes and bipolar disorders. According to Poly-Phen-2 predictions, 82.4% of the ER lumen mutations and 85.7% of the membrane mutations are damaging.

**Conclusion:** We propose mood disorders in Wolfram Syndrome and heterozygous carriers of *WFS1* mutations are the consequence of specific mutations in *WFS1* that alter the structure of wolframin, resulting in intracellular calcium dysregulations and impaired cell signaling, Understanding the effect of *WFS1* mutations on bipolar disorder and schizoprenia is integral to designing clinically targeted treatments for both diseases, which need more specialized treatments.

## Highlights

- Wolfram Syndrome is a rare multi-organ autosomal recessive disease characterized by juvenile diabetes mellitus, diabetes insipidus, optic nerve atrophy, hearing loss, and psychological issues.
- Wolfram syndrome is predominantly caused by mutations in the *WFS1* gene which encodes for wolframin, a protein expressed in most organs including brain, pancreas, lungs, inner ear, and heart.
- Mutations in the gene *WFS1* are associated with psychiatric diseases including bipolar disorder and schizophrenia.
- WFS1 mutations lead to the intracellular calcium dysregulation and impaired cell signaling, which in turn results in mood disorders in subjects with mutations in *WFS1*.
- The majority of the ER lumen and membrane mutations are damaging.

## Introduction

### Wolfram Syndrome Etiology and Diagnostic Criteria

Wolfram Syndrome is an autosomal recessive progressive disease in which patients experience altered physical and psychological functions. The cardinal manifestations include diabetes mellitus coexisting with diabetes insipidus, bilateral optic atrophy, hearing and vision loss along with progressive motor, autonomic and psychiatric abnormalities (1, 2). Wolfram Syndrome is also known by the acronym DIDMOAD (diabetes insipidus, insulin-deficient diabetes mellitus, optic atrophy and deafness) (3, 4). The course of Wolfram Syndrome is progressive, and the prognosis is poor and typically terminal between 30 and 40 years of age. Genetic testing enables reliable diagnosis (1, 3).

Wolfram Syndrome is a rare disease with an estimated prevalence of nearly 30,000 people affected worldwide, categorizing it as an orphan disease. Mutations in the *WFS1* gene are associated with the pathophysiological changes these patients develop [classified by Online Mendelian Inheritance in Man (OMIM #222300)]. *WFS1* encodes for wolframin, an endoplasmic reticulum (ER) associated protein, that is highly expressed in the heart, brain, lungs, inner ear, and pancreas (1, 5). Patients typically are first diagnosed around age six with diabetes mellitus (1). Progressive loss of peripheral vision, color perception, and sensorineural hearing loss usually occurs 4–5 years later (1, 6). Starting in early adulthood, more than half of the patients develop neurological disorders, most commonly manifested as ataxia, seizures, organic brain syndrome, and peripheral neuropathy (1). During this period, psychiatric symptoms, such as depression, mania, and suicidal behavior also occur (7). Brain stem atrophy is also a prominent feature and it is this effect that leads to an early death, secondary to respiratory failure and central apnea (8). Wolfram Syndrome is an aggressive and fatal disease, with the majority of patients with the disease dying before the age of 40.

### Current Treatments

Currently, there is no disease-modifying therapy for Wolfram Syndrome. Management of this disorder is limited to treatment and control of symptoms. Patients typically receive daily insulin injections and dietary modifications to control diabetes mellitus (9). Several other approaches have been used for the management of Wolfram Syndrome. Annual screening for diabetes mellitus, fundus examination, and urodynamic testing are also done (1, 10). There are ongoing clinical trials for disease specific treatments. There is a phase 2 double-blind, placebo-controlled drug directed to evaluate valproic acid (Clinical Trial Number: NCT03717909), a well-known mood stabilizer (11). A second trial is testing dantrolene (Clinical Trial Number: NCT02829268), a drug used to treat malignant hyperthermia (12). The efficacy and safety of these agents for Wolfram Syndrome remain to be established.

### WFS1 Mutations and Neuropsychiatric Disorders

Mutations in the *WFS1* gene have been associated with cognitive abnormalities, in particular psychiatric disorders. These psychiatric disorders include bipolar disorder, schizophrenia, depression, and suicide tendency. Subjects who are either homozygous or heterozygous carriers for mutations in *WFS1* have presented with psychiatric symptoms, ranging from mild to severe (5). The finding of *WFS1* mutations in psychiatric subjects who do not have Wolfram Syndrome led to the suggestion that these mutations are associated with psychiatric symptoms (13) and that mutations in the gene *WFS1* can be biomarkers and predictors for mood disorder (13, 14). Studies have shown that heterozygous carriers of *WFS1* mutations (estimated to be 1% of US population) have a 26-fold increased risk of having a mood disorder (15).

Pathogenic mutations of *WFS1* are found distributed across the entire gene, which is located on chromosome 4p16.1, is approximately 33.4 kilobases in length, and contains eight exons. The gene encodes an 890-amino acid polypeptide associated with the endoplasmic reticulum (16). Although the function of wolframin is unclear, several cellular pathways necessary for normal cell signaling appear to be altered by mutations in *WFS1* including intracellular calcium signaling and its downstream effects, ER stress, and mitochondrial health, neurotransmitter production and release (16, 17). Studies of the role of genetics in psychiatric disorders are challenging because these disorders appear to entail a network of overlapping genes. Similarly, it should be noted that there are both misdiagnoses, nuances, and overlap with other diseases in the characterization of psychiatric disorders. Here we focus on two distinct psychological disorders, bipolar disorder and schizophrenia.

### Bipolar Disorder and Schizophrenia Etiology

Bipolar disorder is a mood disorder that affects ~2% of the human population and is typically characterized by frequent episodes that include mania, hypomania, major depression, and mixed episodes. Bipolar I disorder usually involves a manic/mixed episode, whereas bipolar II must involve a major depressive episode and hypomanic episode, with no requirement for full manic episodes (18). Depressive episodes often become the more prominent aspect of the disorder as patients age. Although the pathophysiology of bipolar disorder is not known, there are environmental, behavioral, cellular, and molecular components that contribute to the risk factors (19). Evidence that bipolar disorder has a genetic basis was shown by the increased susceptibility if other family members have been diagnosed with bipolar disease, where there is a 10-fold increased risk of bipolar disease if the patient has an affected parent (20). Twin studies have shown a 70–85% concordance of disease expression, regardless whether the twins were raised separately or together (20). Schizophrenia is characterized by impairments in high-level cognitive functions such as memory, executive function, and attention along with irregular thought processes, social interaction, and decreased emotional responsiveness. Additional features of schizophrenia include delusions, hallucinations, and difficulties in maintaining long-term relationships. Although little is known about the genetics and molecular bases, genetic studies have shown that schizophrenia and bipolar disorder share a number of the same genes related to the risk of disease (20). Thus, identifying genetic factors provides a guide to potential mechanisms of action.

### Study Aim

In this report, we examined the relationship between reported pathogenic mutations in *WFS1* and psychiatric disorders. We propose that mutations in *WFS1* predict an increased risk of bipolar disorder and schizophrenia. We also note that there are virtually no published studies examining mutations in the N-terminus of *WFS1* which encodes for the primary region of wolframin residing in the cytoplasmic compartment of cells and represents over 20% of the protein. Combining results from all these studies will assist in the understanding of the pathophysiology of both Wolfram Syndrome and psychiatric disorders.

## Methods

### Searching Strategy and Study Selection

**Figure 1:**
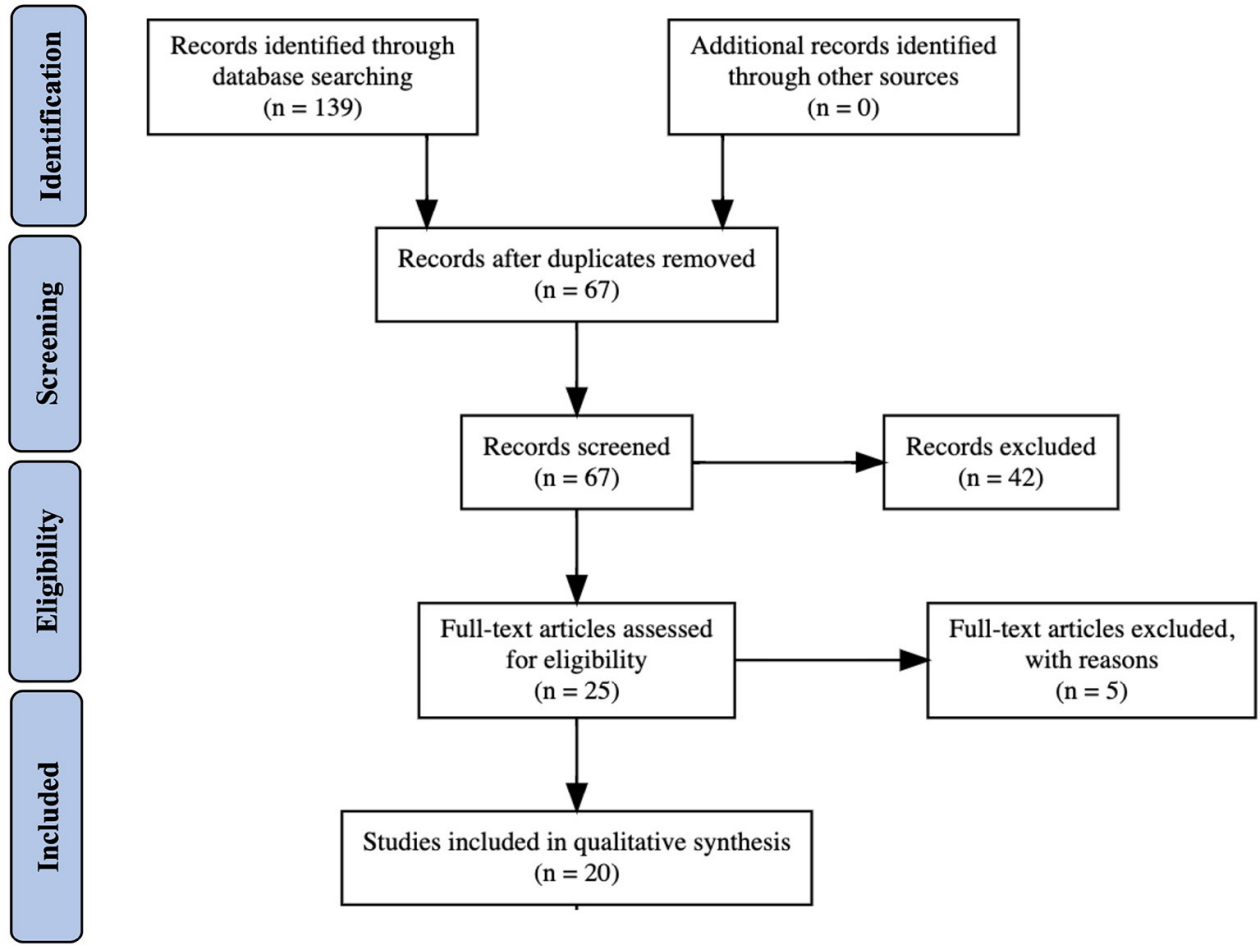
Flow diagram of papers searched and selected for final report.

Comprehensive literature searches and analyses were done to compile available data regarding Wolfram Syndrome and heterozygous carriers of *WFS1* mutations with psychiatric disorders (Figure 1). Databases searched included PubMed Central, MEDLINE, Google Scholar, and Bookshelf. Within all databases, the search terms “Wolfram Syndrome and Mood Disorders,” “N-terminus mutations in *WFS1*,” and “*WFS1* mutations” AND (bipolar disorder, mood disorder, depression, or psychiatric disease) were used. Papers (ranging from 1998 to 2020) were selected after reading the abstract and determining relevancy to the objective. The aim was to determine if particular *WFS*1 mutations found in Wolfram Syndrome patients were associated with psychiatric symptoms regardless of whether the patient exhibited classic symptoms of Wolfram Syndrome.

Published papers that ranged from small patient studies to in depth genomic analyses were gathered. The literature was mined for relevant patient data and identification of mutations in *WFS1*. In addition, the references of each paper were examined for any other relevant papers. We excluded any study that does not address the full protein sequence of *WFS1*. However, the paucity of studies that examined mutations in the N-terminus of *WFS1* highlights that mutations in the first 310 residues of the protein are underrepresented.

### Data Synthesis Through *in-silico* Evaluation Software

In order to select the relevant mutations, the *WFS1* amino acid sequence was run through SNAP2 (screening for *n*on-acceptable polymorphisms), PolyPhen-2 (Polymorphism Phenotyping v2), and PROVEAN (Protein Variation Effect Analyzer), all of which are software that predict the functional effects of sequence variants using statistical methods (21, 22).

The first program used was SNAP2 (23). SNAP2 is based on a learning system known as a neural network and uses factors such as multiple sequence alignment, predicted secondary structure, and solvent accessibility to determine the functional effect of mutations. SNAP2 scores the mutations on a scale from −100 to 100, where −100 is very likely no effect and +100 is strong likelihood of effect (24). Any values above 0 indicate that there is a structural effect to the protein. SNAP2 also gives a percent expected accuracy for their predictions about mutation score and functional effect. Therefore, only mutations that had a predicted effect score >0 and a percent expected accuracy >50% were compiled in this study.

Next, the chosen mutations from the SNAP2 analysis were sent through PolyPhen-2. PolyPhen-2 predicts the functional effects of sequence variants using both structural and evolutionary based data. PolyPhen-2 sequentially builds a conservation profile and determines the probability of the protein being damaged as a result of the missense mutation. PolyPhen-2 determines each mutation to be benign, possibly damaging, or probably damaging.

The final program implemented was PROVEAN. PROVEAN also predicts the functional effect of amino acid substitutions, insertions, and deletions. PROVEAN's “alignment-based score” quantifies the effect on protein expression, structure, and function based on amino acid changes. PROVEAN scores the mutations and any mutation with a value <-2.5 is considered deleterious. Above −2.5 is considered neutral.

It is difficult to characterize a certain psychiatric symptom as a definite indicator of a particular disorder. Thus, our investigation of mutations in the gene *WFS1* incorporated data that include psychiatric symptoms associated with bipolar disorder and schizophrenia, even if those patients were not formally diagnosed with one of the disorders. To take into consideration lack of diagnosis and misdiagnosis, all patients with the following symptoms were considered: suicide tendency, suicide, depression, major depressive disorder, schizophrenia, bipolar disorder, manic episodes, hallucinations, and general psychiatric illness. As little is known about the cellular and neurological function of either wolframin or *WFS1*, there is not an abundance of data surrounding its connection to psychiatric disorders, especially as psychiatric disorders are not a defining characteristic of the disease (1). In total, approximately 2,392 patients were included in these collective studies.

### Statistical Analysis

Fisher's Exact Tests were performed to investigate: (i) the correlation between mutation location and the predicted PolyPhen-2 score and (ii) the correlation between mutation location and psychiatric disorders. A *P*-value lower than 0.05 was considered statistically significant, and correlation indices such as Cramer's V and Contigency Coefficient were computed.

In order to illustrate the correlation of psychiatric phenotypes with the different locations, we categorized the psychiatric disorders into 5 main groups:

- Aggression, hallucinations, general psychiatric illness and psychiatric problems
- Depression, depression with therapy and major depression
- Manic episodes and bipolar disorder
- Schizophrenia
- Suicide.

## Results

The first round of literature search yielded over one hundred possible *WFS1* mutations that were associated with mood disorder. In order to refine this list to a more concise and accurate list, SNAP2 was used to determine the predicted effect and accuracy on the protein structure and amino acid sequence [UniProtKB—O76024 (WFS1_HUMAN)]. The predicted protein structure was previously generated by I-TASSER (25). As the experimental validation of the wolframin structure is not yet established, it was necessary to utilize this molecular prediction to identify the likely location of the mutations on the protein.

**Figure 2:**
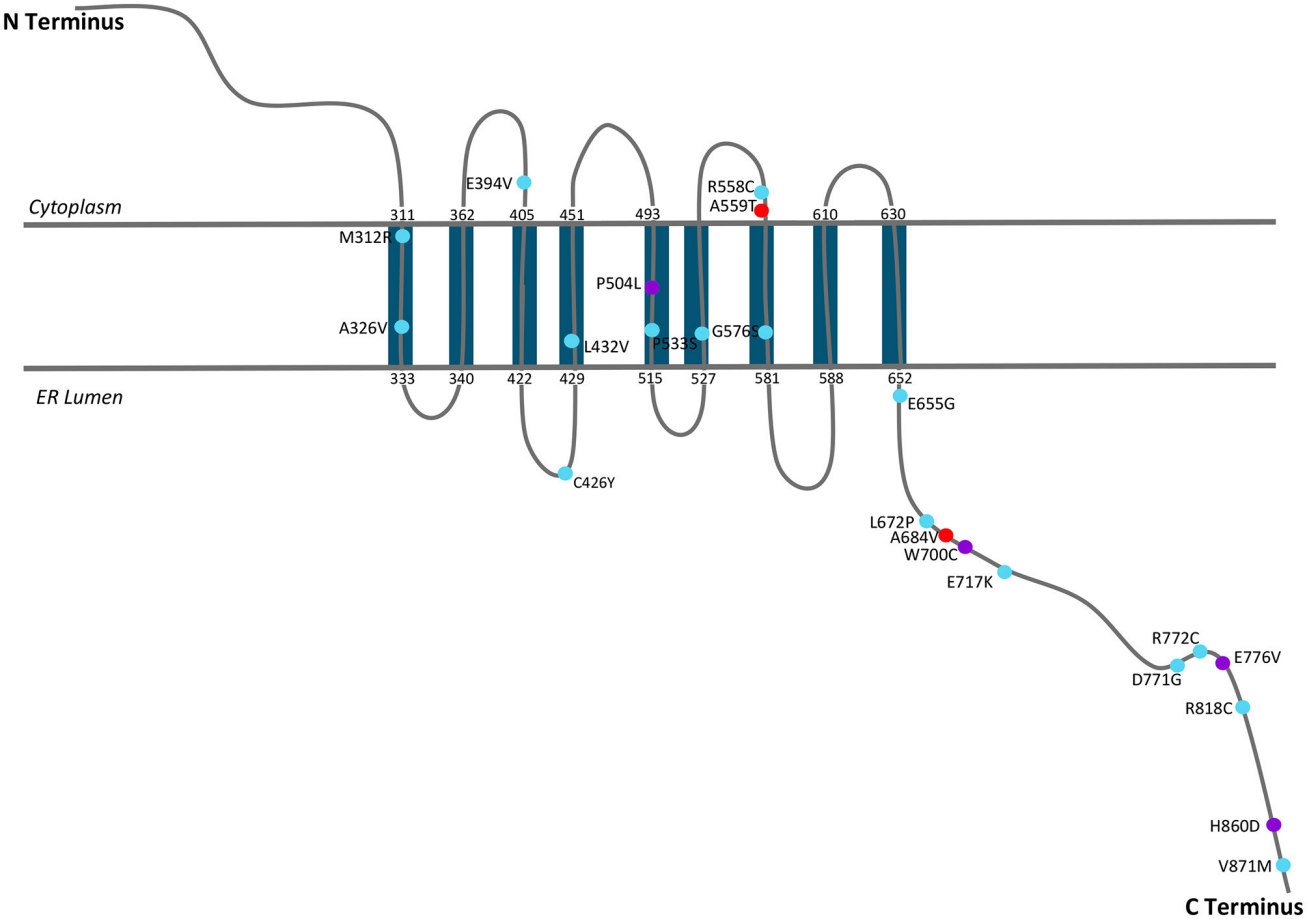
A schematic of *WFS1* with mutations that have been shown to be associated with mood disorder. Figure was adapted from 2014 Matsunaga et al. mutation map (27).

Wolframin is a multi-pass transmembrane protein associated with the endoplasmic reticulum (ER) (16, 17, 26), where the N-terminus is cytoplasmic and the C-terminus resides in the lumen of the ER. The identified mutations were highlighted on the proposed folding structure of wolframin (Figure 2). This location and orientation are appropriate for a protein that regulates calcium signaling (17). This mutation map demonstrates the many reported C-terminal mutations, but lack of known mutations in the N-terminus. The absence of published reports on mutations in the N-terminus was unexpected. Although the mutations associated with Wolfram Syndrome appear to span the entire protein, the most frequently identified mutations associated with psychiatric symptoms are A559T and A684V, both of which are predicted to be localized in extra-membrane regions of the protein. Several mutations in non-Wolfram Syndrome patients (R772C, E717K, and M312R) occurred four unique times in different data sets, suggesting that these are important positions for the function of wolframin. When a defined structure for wolframin is available, it will be possible to confirm the location of mutational hotspots in the protein. From the present information, we identified regions of interest which included mutations that had multiple reports in the C-terminus (residues 672–717 and 771–776) and at the interface between the membrane and the ER lumen (A326, L432, P533, G576).

**Figure 3:**
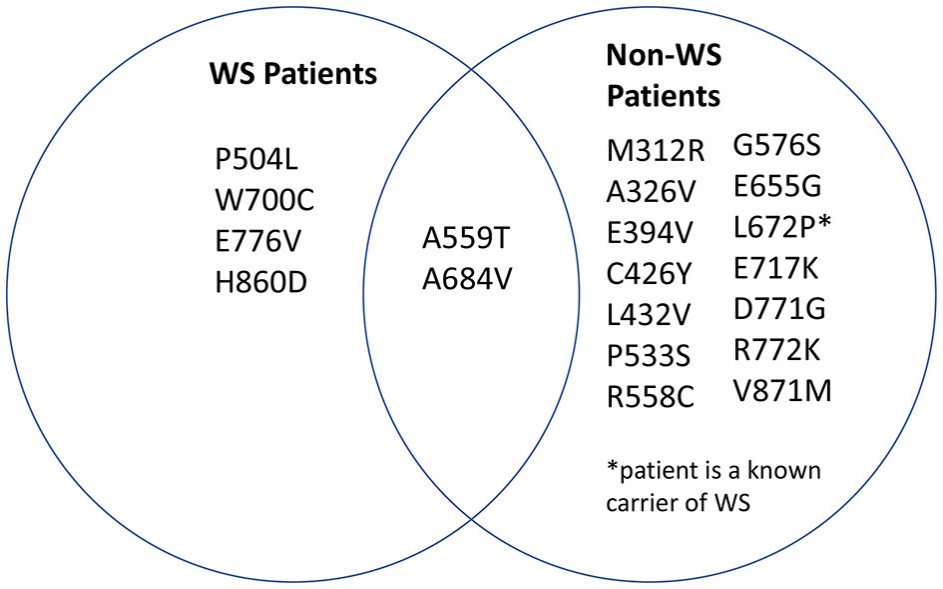
Distribution of mutations associated with psychiatric symptoms. *WFS1* mutations associated with mood disorder were categorized into two groups: mutations identified in Wolfram Syndrome patients and mutations identified in heterozygous carriers of *WFS1* mutations.

We then sought to determine which mutations found in our search were associated with Wolfram Syndrome patients and which mutations were associated with heterozygous carriers of *WFS1* mutations. A Venn diagram was generated in order to visualize the overlap (Figure 3). Note that two mutations in wolframin (A559T and A684V) were present in both Wolfram Syndrome and heterozygous carriers of *WFS1* mutations in multiple cases. A559T is associated with manic episodes and major depression (15, 28, 29), and A684V is associated with depression and hallucinations (30–32) in both populations.

**Figure 4:**
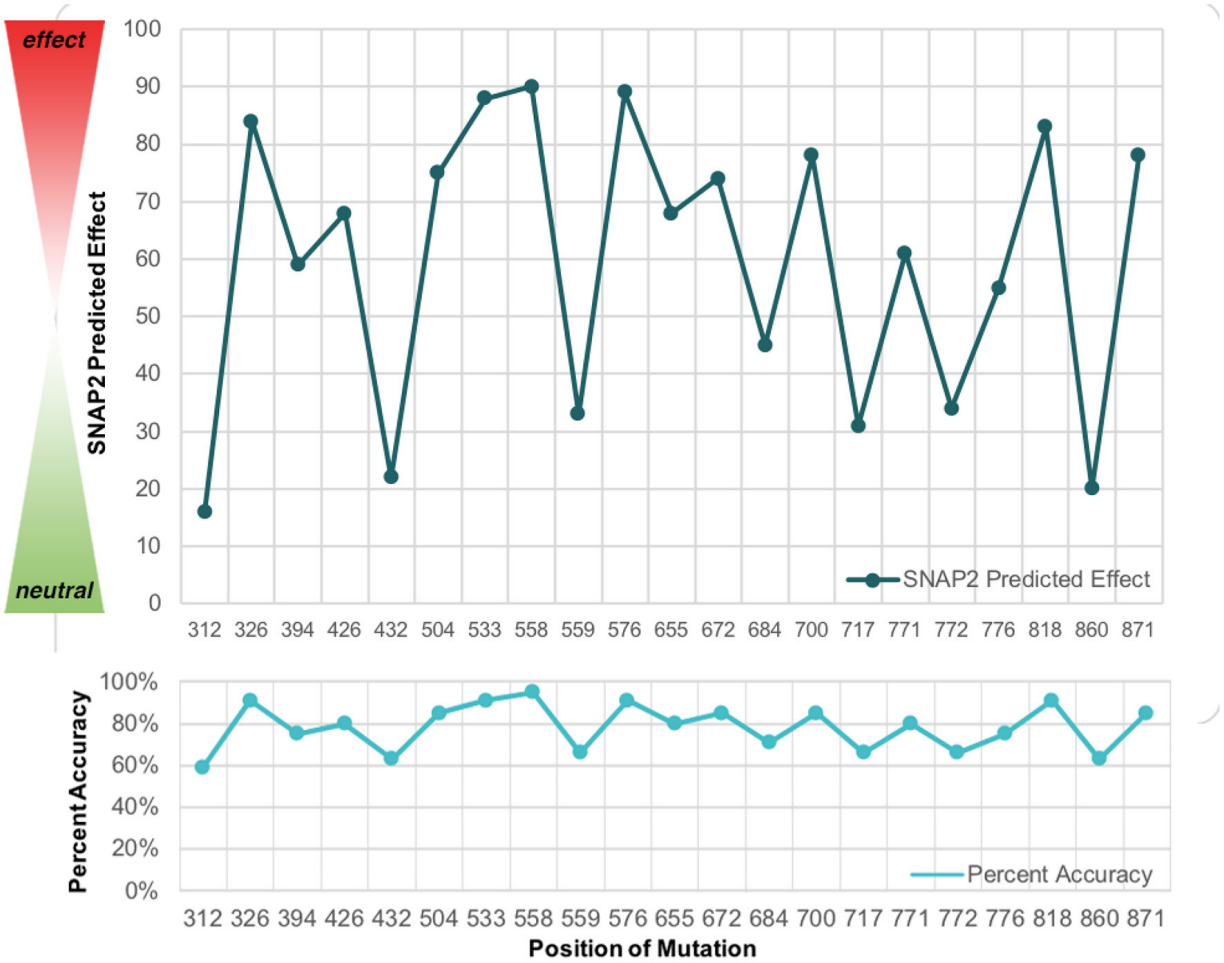
The SNAP2 predicted effect of mutations in WFS1 in with the SNAP2 predicted accuracy for each mutation's impact. The closer to 100 a particular mutation is, the greater impact it has on wolframin structure and function. Note how the pattern for predicted effect and percent accuracy mirror each other.

Subsequently, the SNAP2 predicted effect values were plotted in comparison to the SNAP2 predicted accuracy values (Figure 4). The SNAP2 results were most accurate when considering mutations with extreme impact on protein structure and function. For example, W700C is a mutation which has an extreme impact on the structural integrity of wolframin and had a high SNAP2 predicted effect score and high percent accuracy, whereas a mutation such as M312R has a low SNAP2 predicted effect score and a low percent accuracy. Patients with mutations at W700 have severe manifestations of Wolfram Syndrome because the mutation W700X causes truncation of the majority of the C-terminus (10). Also, patients with the mutation W700C have been found to have severe mood disorder with the mutation (9, 33). The psychiatric symptoms associated with W700X were not reported. Patients with M312 mutations had milder symptoms of Wolfram Syndrome but had psychiatric symptoms (34). Altered protein structure can be easily detected, especially when the protein is truncated. The degree of alteration in protein structure appears to correlate with the impact on functions related to Wolfram Syndrome. The mutations to *WFS1*, especially those to W700, should be examined further for effects on wolframin structure that lead to both medical and psychiatric downstream events.

The mutations evaluated with SNAP2 were further analyzed using PROVEAN and PolyPhen-2. The effect and accuracy of each mutation was examined, and the values were organized into a chart with the following information for each mutation: name and position, associated mood disorder, SNAP2 predicted effect score/expected accuracy (%), PolyPhen-2 predicted effect, and PROVEAN predicted effect score (Table 1). The PROVEAN and PolyPhen-2 results mirror those of the SNAP2 results.

**Table 1:** Mutations in wolframin listed with their associated symptoms, SNAP2 predicted effect score/SNAP2 predicted percent accuracy, PolyPhen-2 rating, and PROVEAN rating/PROVEAN numerical score.

**Mutation**	**Associated psychiatric disorder (s)**	**SNAP2 score for predicted effect/expected accuracy**	**PolyPhen-2**	**PROVEAN (cut off at −2.5)**	**References**
M312R	Schizophrenia	16/59%	Benign	Neutral/−1.989	(2–4)
A326V	Suicide	84/91%	Probably Damaging	Neutral/−0.556	(2–4)
E394V	Suicide	59/75%	Probably Damaging	Deleterious/−6.106	(5–7)
C426Y	Major depression	68/80%	Benign	Neutral/1.805	(7–9)
L432V	Major depression, Schizophrenia	22/63%	Probably Damaging	Neutral/−2.265	(3, 9)
P504L	Depression, psychiatric problems	75/85%	Probably Damaging	Deleterious/−3.386	(3, 10)
P533S	Suicide	88/91%	Probably Damaging	Deleterious/−7.923	(6)
R558C	Schizophrenia	90/95%	Probably Damaging	Deleterious/−7.134	(8)
A559T	Manic episodes, major depression	33/66%	Benign	Neutral/−0.961	(10–12)
G576S	Schizophrenia	89/91%	Possibly Damaging	Neutral/−1.796	(9)
E655G	Suicide	68/80%	Probably Damaging	Deleterious/−3.488	(5)
L672P	General Psychiatric Illness, Major depression	74/85%	Probably Damaging	Deleterious/−4.742	(6, 12)
A684V	Depression, hallucinations	45/71%	Probably Damaging	Neutral/−2.203	(13)
W700C	Depression and aggression	78/85%	Probably Damaging	Deleterious/−12.937	(6, 8, 10, 11)
E717K	Major depression	31/66%	Probably Damaging	Neutral/−1.892	(4, 9, 13, 14)
D771G	Schizophrenia	61/80%	Probably Damaging	Deleterious/−3.942	(6, 8, 15)
R772C	Schizophrenia	34/66%	Probably Damaging	Deleterious/−4.222	(3, 6, 7, 9)
E776V	Depression requiring therapy	55/75%	Probably Damaging	Deleterious/−6.223	(6, 10, 15)
R818C	Schizophrenia, Bipolar disorder	83/91%	Probably Damaging	Deleterious/−3.748	(6, 10, 11)
H860D	Depression requiring therapy	20/63%	Probably Damaging	Neutral/−1.975	(6, 10)
V871M	Schizophrenia, Bipolar disorder	78/85%	Benign	Neutral/−0.196	(3, 13)

**Figure 5:**
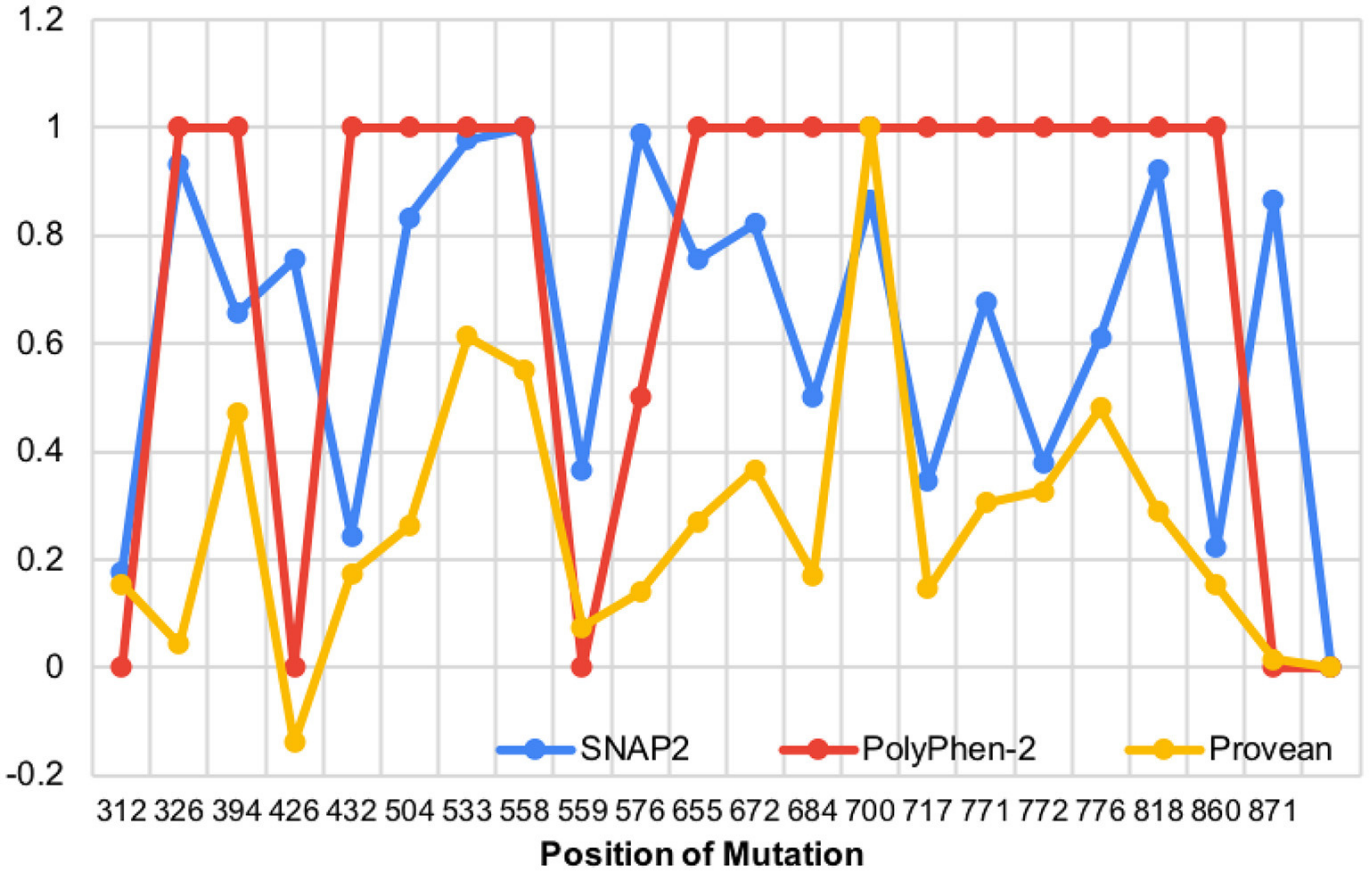
The impact of WFS1 mutations on protein structure and function based on SNAP2, PolyPhen-2, and PROVEAN data (data points were normalized to highest value in each data set).

We then aligned the magnitude of every mutation's impact along the protein (Figure 5). It is important to note that there are no data regarding the N-terminal mutations in *WFS1* and mood disorder, thus our analysis of mutations begins after the N-terminus, and the X axis starts at residue position 300. The mutations which were detected across all three platforms to be particularly impactful (in order from most to least impactful) were W700C, P553S, and R558C. W700C was given a PROVEAN of−12.937, which is notably larger than the ratings of P533S and R558C (−7.923 and −7.134, respectfully). Note that P533S and R558C mutations have only been detected in heterozygous carriers of *WFS1* mutations. Thus, these mutations (P533S and R558C) may be points of interest for psychiatric disease because of their location in the middle of the protein, not the N- or C- termini (Table 1). This central location may allow wolframin to function relatively normally and cause solely psychiatric symptoms as opposed to physical symptoms like those associated with Wolfram Syndrome patients.

Over 29 mutations, we observed that 4 mutations occurred in the cytoplasm, 17 in the ER lumen, and 7 in the membrane. We found a statistically significant correlation between the mutation location and the predicted PolyPhen-2 score (Cramer's V = 0.40, Contigency Coefficient = 0.49, *P*-value in Fisher's Exact Test = 0.02). The data showed that 82.4% (14/17) of the ER lumen mutations and 85.7% (6/7) of the membrane mutations are predicted as “probably damaging” according to Poly-Phen whereas 60% (3/4) of cytoplasmic mutations are predicted to be “benign” (Table 2). This finding suggests that if a mutation occurs in the cytoplasm it is more likely to be benign; conversely if the mutation occurs in the lumen or in the membrane, then it is more likely to be damaging.

**Table 2:** Relationship between mutation locations and PolyPhen-2 predicted effect.

	**PolyPhen-2 predicted effect**, ***N*** **(%)**		
**Location (*N*)**	**Benign**	**Possibly damaging**	**Probably damaging**
Cytoplasm (5)	3 (60%)	0 (0%)	2 (40%)
ER lumen (17)	3 (17.6%)	0 (0%)	14 (82.4%)
Membrane (7)	0 (0%)	1 (14.3%)	6 (85.7%)

Table 3 reports the distribution of the mutations associated to the psychiatric disorder's groups across different locations. About 35% (10/29) and 27.6% (8/29) of the total mutations are associated with the “Depression, depression with therapy and major depression” group and with the “Schizophrenia” group, respectively.

**Table 3:** Distribution of the mutations associated to the psychiatric disorder groups across different locations.

	**Location**, ***N*** **(%)**		
**Psychiatric disorders group (*N*)**	**Cytoplasm**	**ER lumen**	**Membrane**
Aggression, hallucinations, general psychiatric illness and psychiatric problems (4)	0 (0%)	3 (75%)	1 (25%)
Depression, depression with therapy and major depression (10)	1 (10%)	7 (70%)	2 (20%)
Manic episodes and bipolar disorder (3)	1 (33.3%)	2 (66.7%)	0 (0%)
Schizophrenia (8)	2 (25%)	4 (50%)	2 (25%)
Suicide (4)	1 (25%)	1 (25%)	2 (50%)

The analysis did not show a statistically significant correlation between a certain psychiatric disorders group and mutation location (Cramer's V = 0.29, Contigency Coefficient = 0.38, *p*-value reported by the Fisher's Exact Test = 0.77). The majority of the mutations, indeed, occurred in the ER lumen, regardless the psychiatric disorders groups. In patients with schizophrenia there are two mutations in cytoplasm, 4 in the ER lumen and 2 in the membrane. In patients with manic episodes and bipolar disorders one cytoplasmic and 2 ER lumen mutations were reported.

## Discussion and Conclusion

Wolfram Syndrome is a rare multisystem disease characterized by juvenile onset diabetes mellitus, vision and hearing loss, and psychiatric disorders. There is no definitive diagnostic tool nor disease-modifying tool for Wolfram Syndrome-related mood disorders. Presently, there is little known about the protein wolframin and gene *WFS1*. Similarly, a coherent molecular or genetic understanding of mood disorders is still needed. The goal of this literature analysis was to determine whether there are hotspots in wolframin that could link mutations in the protein associated with Wolfram Syndrome to mood disorders such as bipolar disorder and schizophrenia. Wolfram Syndrome has previously been used as a model for diabetes, hearing and vision loss (1, 3). Using alterations in the wolframin associated pathway as a model for mood disorder is a novel application. Here, we analyze the relationship between *WFS1* mutations and psychiatric disorders, mainly schizophrenia and bipolar disease.

Relevant to this work, there is a growing interest in the field of genetic mutations related to psychiatric diseases. For example, evidence suggests the existence of mutations that are causative to autism spectrum disorder (35), Tourette's Syndrome (36), attention-deficit/hyperactivity disorder (37), and Huntington's Disease (38). The correlation we observed between *WFS1* mutations and psychiatric symptoms is consistent with these previous findings in other diseases.

Our findings support the proposal that specific mutations in *WFS1* are associated with mood related disorders in both Wolfram Syndrome patients and heterozygous carriers of *WFS1* mutations. Through a literature-based analysis, several mutations were found to be disease relevant using the SNAP2 platform. When the location of these mutations is considered (Table 1), there is evidence that there are hot spots even though mutations were found throughout the protein.

Studies that have examined the N-terminal region of *WFS1* have indicated that this region includes many “loss of function” mutations (1, 3). These mutations are often truncations or frameshifts which completely alter the remainder of the protein and are associated with the most detrimental forms of Wolfram Syndrome (1). More information describing the consequences of these N-terminus mutations is needed. We expect that there is a high incidence of mood disorders in individuals who carry mutations in this region, in both Wolfram Syndrome and heterozygous carriers of *WFS1* mutations.

Mutations in heterozygous carriers of *WFS1* such as R772C, E717K, and M312R, occurred four unique times in different data sets throughout this search, which suggests that they are important positions. A next step would be to perform mutagenesis of wolframin in cell and animal models of Wolfram Syndrome to assess how the mutations affect mRNA and protein stability *in vitro* and *in vivo*. R772C and E717K are both in the C-terminus, which is associated with severe Wolfram Syndrome and psychiatric illness. It will be important to learn if these residues are localized near W700 in the 3D folded structure of wolfram, another residue associated with severe disease. In contrast, M312R is closer to the N-terminus on a transmembrane loop predicted to be close to the cell cytoplasm. Interestingly, this location at the interface between membrane and cytoplasm may be relevant as other mutations associated with psychiatric symptoms were found there (A559T, E394V and R558C). In addition, the mutation R558C has been found to cause a mild form of Wolfram Syndrome and is associated with risk for type 2 diabetes in the Ashkenazi Jewish population (39). This group of individuals provides a controlled group to study the effects of the low impact mutation both physically and psychologically. Once the wolframin structure is known, interactions among these sites will be determined. Future efforts and experiments will extend these findings and assist our understanding about Wolfram Syndrome and its related psychiatric symptoms.

The mutated protein wolframin or one of its binding partners could be used as a potential druggable target for associated mood disorders. For example, the interaction between wolframin and Neuronal Calcium Sensor 1 (NCS1) (40), a calcium binding protein, has potential as a drug target (17). NCS1 expression level is low in fibroblasts isolated from patients with Wolfram Syndrome patients compared to control (16) and is highly expressed in patients with bipolar disorder and schizophrenia, specifically with an up-regulation in the prefrontal cortex (41). Ideally, drugs used to treat mood disorder could also treat Wolfram Syndrome, including related symptoms such as diabetes and brain stem atrophy. One potential drug used for bipolar disease, lithium, can enhance neuronal health (42), but there are long-term negative side effects on overall patient health. Therefore, lithium does not appear to be a compelling candidate for long-term treatment for Wolfram Syndrome. In contrast, valproate, a drug used as an anticonvulsant and for bipolar disease (11), is being evaluated as a treatment for Wolfram Syndrome patients (Clinical Trial Number: NCT03717909). Ibudilast, a PDE4 inhibitor and NCS1-binding drug, is a known neuroprotective agent and was shown to restore cellular functions in *WFS1*-deficient beta cells (17). To identify and optimize additional candidates as effective drugs for Wolfram Syndrome, it is important to uncover as much as possible about the molecular bases of this rare disease.

The findings of this report should be interpreted considering several limitations. Most importantly, knowledge of N-terminus mutations in Wolfram Syndrome patients is still limited and the availability of literature discussing this specific topic is sparse. In addition, information about heterozygous carriers of *WFS1* mutations is also under reported. These limitations establish significant obstacles to find and select relevant studies to substantiate our findings. Therefore, we formulated our search with a focus on schizophrenia and bipolar disease, as opposed to generally searching for every term associated with mental illness. Because this report was not conducted as a formal meta-analysis, a quality assessment strategy could not be applied. We consider this report as an exploratory study intended to lay the groundwork for a more comprehensive research study in the future, which could hopefully include further information regarding psychiatric symptoms and N-terminal *WFS1* mutations. In addition, with a debilitating disease such as Wolfram Syndrome, there is a chance that some of the psychiatric disorders identified in patients were an effect of living with a complex and incurable disease and not caused directly by the gene mutated in Wolfram Syndrome. Although we completed the review with these ambiguities in mind, understanding the distinction between cause and effect of mood disorders in particular is an area of research which needs further study.

Even though it is hypothesized that the location of *WFS1* mutations would correlate with psychiatric disorders, the results of our statistical analysis failed to confirm this hypothesis. There are several explanations for these results. For example, the wide spectrum of psychiatric symptoms reported in Wolfram Syndrome would require larger sample sizes and more evident phenotypes to adequately test for a correlation. Furthermore, there is variation in both the prevalence and the clinical assessment of psychiatric symptoms in different regions the world. Additionally, psychiatric manifestations are known to be influenced by environmental factors.

When we examined Poly-Phen 2 predictive scores, we found that most of membrane and ER lumen mutations are damaging. As ER function is intimately related to the DNA damage response, and given the accumulating data supporting the hypothesis that ER and membranal wolframin mutations significantly induce psychiatric illness, we suggest that altered calcium homeostasis due to decreased NCS1 protein abundance is a contributing factor for the impaired cell signaling leading to abnormal neuronal function.

Current treatments for both Wolfram Syndrome and mood disorders are limited. Often, the treatments are centered around symptom management as opposed to addressing the underlying mechanism responsible for the disease. In the case of Wolfram Syndrome related-mood disorder, patients are typically seen when their disease has progressed to an unmanageable point. Although Wolfram Syndrome is usually detected early, the disease progresses quickly, and leaves little time to reverse the damage at the cellular level. As mood disorders are complex, management may need to be personally tailored, and thus there is a demand for a targetable and druggable pathway for these conditions. Further research on the pathways and mutations underlying Wolfram Syndrome, particularly the N-terminus of *WFS1*, is crucial to provide early diagnoses and effective therapeutics.

**Figure 6:**
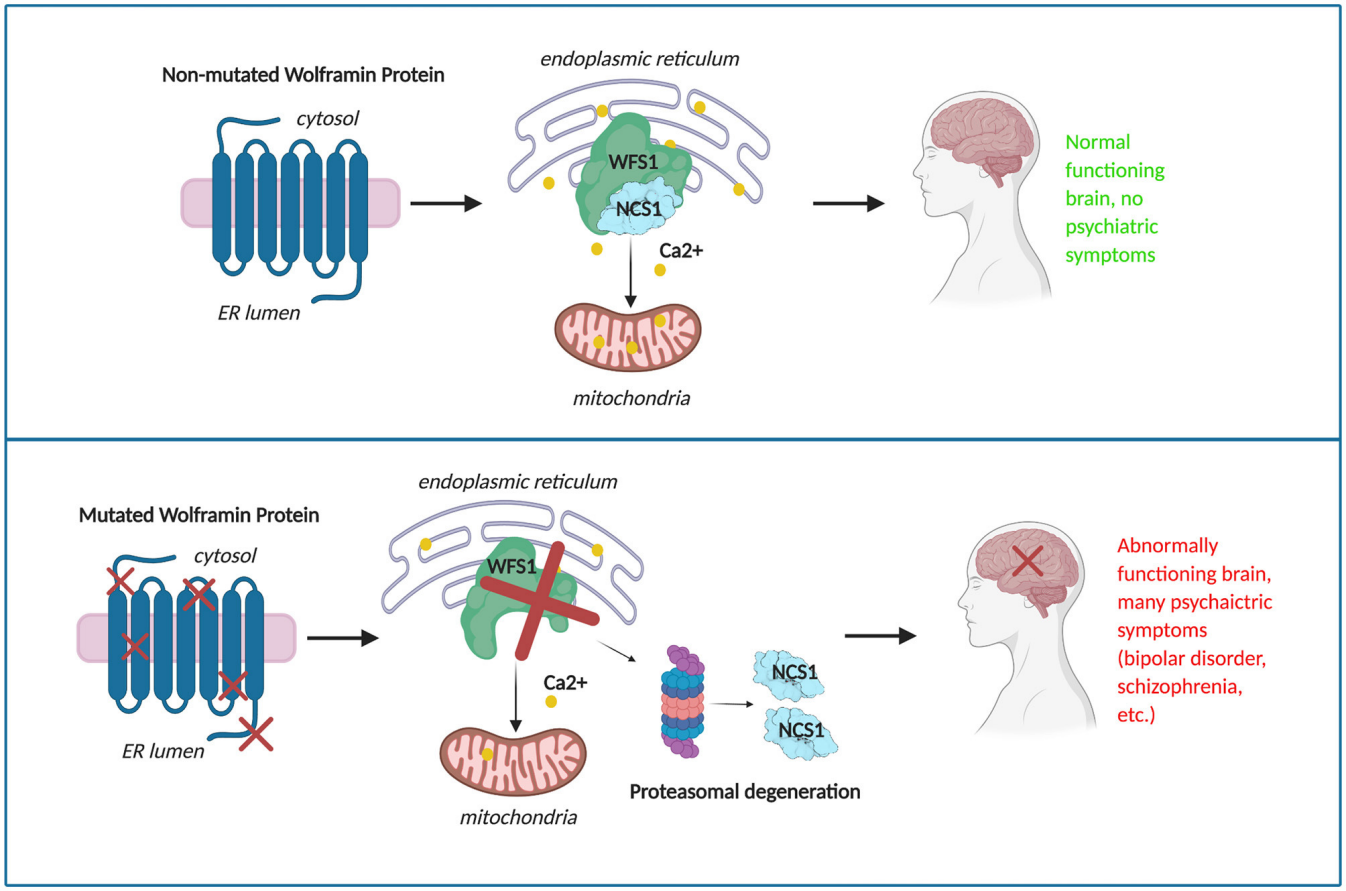
The potential progression of mood disorder in a given patient due to mutations in wolframin.

In conclusion, *WFS1* mutations have shown impactful change in the structure of wolframin, leading to impaired calcium signaling and cell survival (17). These changes are expected to result in mood related disorders in Wolfram Syndrome and heterozygous carriers of *WFS1* mutations (Figure 6). Understanding the effect of *WFS1* mutations on psychiatric disease is an important challenge that will aid to development of targeted treatments for both Wolfram Syndrome and mood disorders.

## Data Availability Statement

The raw data supporting the conclusions of this article will be made available by the authors, without undue reservation.

## Author Contributions

SM and BE conceived the project. SM and EI analyzed all data. SM wrote the first draft of the manuscript. Statistical analysis was done by ID. All authors edited the manuscript and agreed to the final manuscript.

## Funding

This work was supported by a grant from the National Institutes of Health: P01DK057751 (BE) and a generous gift from an anonymous donor.

## Author Disclaimer

The views expressed in this article are those of the authors and do not necessarily reflect the position or policy of the Department of Veterans Affairs or the United States government.

## Conflict of Interest

BE is a founder of Osmol Therapeutics, a company that is targeting NCS1 for therapeutic purposes. ID is a biostatistician at the West Haven, CT Cooperative Studies Program Coordinating Center, VA Office of Research and Development. The remaining authors declare that the research was conducted in the absence of any commercial or financial relationships that could be construed as a potential conflict of interest.

## Publisher's Note

All claims expressed in this article are solely those of the authors and do not necessarily represent those of their affiliated organizations, or those of the publisher, the editors and the reviewers. Any product that may be evaluated in this article, or claim that may be made by its manufacturer, is not guaranteed or endorsed by the publisher.

## Abbreviations

DIDMOAD: diabetes insipidus, insulin-deficient diabetes mellitus; optic atrophy and deafness
ER: endoplasmic reticulum.
